# Work ability and work functioning: measuring change in individuals recently returned to work

**DOI:** 10.1007/s00420-019-01400-z

**Published:** 2019-01-17

**Authors:** A. van Schaaijk, K. Nieuwenhuijsen, M. H. W. Frings-Dresen, J. K. Sluiter

**Affiliations:** Coronel Institute of Occupational Health, Amsterdam Public Health research institute, Amsterdam UMC, University of Amsterdam, Meibergdreef 9, PO Box 22660, 1100 DE Amsterdam, The Netherlands

**Keywords:** Minimally clinical important difference, Occupational physician, Return to work, Smallest detectable change, Work Ability Score

## Abstract

**Purpose:**

To assess: (1) whether work ability and work-functioning instruments can detect relevant changes in their respective parameters following a return to work (RTW) and (2) what proportion of those returning to work show changes in their work ability and work functioning.

**Methods:**

A total of 1073 workers who returned to work after at least 2 weeks of sick leave were invited to fill out three questionnaires in the first 8 weeks after RTW. These consisted of an appraisal of general, physical, and mental/emotional work ability (scores 0–10) and a work-functioning questionnaire (scores 0–100). Minimal Important Change (MIC) was defined to determine the proportion of people, whose scores had changed at weeks 5 and 8 following RTW. The Smallest Detectable Change (SDC) was determined to put the MIC in perspective of measurement error.

**Results:**

Of all participants, 235 were eligible for the analysis. All MIC values were below the SDC and thus not suitable for use. The SDC for work ability was 2.2 and 19.9 for work functioning. In the first 5 weeks after RTW, 10–15% showed a relevant, measurable improvement in work ability, and work functioning based on the SDC margins.

**Conclusions:**

Both instruments were unable to identify change after RTW adequately. We can conclude that 10–15% of individuals showed improvement in work ability and work functioning in the first 5 weeks after RTW when SDC is used.

## Introduction

Work ability and work functioning are important parameters in a working population. Both concepts relate to a workers’ assessment of being capable of carrying out the work given his or her health condition. Work ability is a generic term that includes all aspects of the ability to work at once, and work functioning focusses on several domains that have influence on the functioning of a worker. When workers are signed off sick, the work ability and work functioning are affected. The goal for occupational physicians and other occupational health professionals is to allow them to return to work (RTW) quickly while optimizing their health.

Several strategies have been developed to improve the RTW of workers returning from sick leave (Hogelund et al. 2010; Schaafsma et al. 2013; Schneider et al. 2016). In many of these strategies, an employee returning from an extended sick leave will be reintroduced back into the workplace in steps, e.g., for periods of fewer hours, in a graded RTW programme or with modified requirements of workplace activities (Hogelund et al. 2010; Viikari-Juntura et al. 2012; Viikari-Juntura and MacEachen 2015). Central to these strategies is that the functioning of the employees on RTW—their performance, productivity, quality, quantity, and capacity—must be sufficient in those hours and activities. When working fewer hours, the employee has more time to recover from work before the next workday starts. With modified activity, the worker executes only the tasks that they can fulfill given their health situation. It is likely that, in the weeks following the RTW, an individual experiences a gradual increase in work ability and work functioning (Franche and Krause 2002). If this increase can be identified, the occupational health professional or manager can intervene if improvement does not occur. This can prevent recurrent sick leave by adjusting the work according to the workload capacity growth or loss.

For a successful RTW, a worker needs sufficient work ability. It is important to monitor individual work ability in the RTW process (Ilmarinen et al. 2005). Different instruments to assess work ability and work functioning can be found in the literature (Boezeman et al. 2015b; Burton et al. 1999; El Fassi et al. 2013; Endicott and Nee 1997; Kessler et al. 2003; Koopman et al. 2002; Lerner et al. 2001, 2003; Reilly et al. 1993; Shikiar et al. 2004; van Roijen et al. 1996). Two of these instruments are the appraised work ability and composite work-functioning instrument (Boezeman et al. 2015b; El Fassi et al. 2013). The appraised work ability tool is a single-item self-appraisal of workers’ current work ability compared to their lifetime best, derived from the Work Ability Index (Tuomi et al. 1998). The composite work-functioning tool is a 49-item domain-based work-functioning questionnaire. For this study, these two instruments were chosen, because the Work Ability Score is a single-item question and may be useful for quick assessment, while the work-functioning score can add valuable information on the different domains that need attention for the RTW process.

It is unknown whether the RTW process is linked to a relevant improvement in work ability and work functioning. We expect that after RTW, work participation gradually returns to the original level or a new maximum, and should thus be associated with a positive change in work ability and work functioning over time. Currently, occupational physicians in The Netherlands rarely use these existing instruments to assess the quality of RTW after absenteeism. One of the reasons may be that the use of these instruments in this context is not yet tested, and the magnitude for relevant change in these instruments is not yet established. Besides this, the time frame in which any relevant change should have occurred after RTW still needs to be defined. Quality of RTW is assessed as the extent to which an employee can fully function again based on his health and the requested work demands. For these instruments to be able determine any relevant changes following an RTW, and establish the optimal time frame in which to deploy the instruments, the trajectory of work ability and work functioning after a period of absenteeism needs to be studied.

It is unknown if the existing instruments for measuring work ability and work functioning are able to detect a change in work ability and work functioning in the early weeks after absenteeism. Therefore, the primary goal of this study is to assess whether these two instruments to monitor work ability and work functioning can detect a change over the first 2–8 weeks of RTW following absenteeism of at least 2 weeks. This leads to the first research question:

### *Q1*

To what extent are the work ability and work-functioning instrument able to detect relevant change after RTW irrespective of the origin of absence?

Subsequently, we aim to use both instruments (the appraised work ability tool and composite work-functioning questionnaire) to determine the trajectories of both work ability and work functioning in the early weeks after RTW. If we can determine what a relevant change is for both instruments, we can determine the optimal time to administer the questionnaires. Towards this end, we assess at what time after returning to work the majority of workers have made a relative improvement in work ability or work functioning. We envision that this will allow occupational professionals to monitor RTW and have the ability to identify—and subsequently intervene—if improvement in work ability or work functioning does not occur. This leads to the second research question:

### *Q2*

What proportion of employees show a relevant change (as determined in the previous research question) in work ability and work functioning, in the first 8 weeks after RTW?

## Study population and methods

### Ethics statement

The research was conducted in accordance with the declaration of Helsinki (WMA 2013). The research proposal was approved by the Medical Ethical Committee of the Academic Medical Center, who judged that a comprehensive evaluation was not required, since this study was not subject to the Medical Research Involving Human Subjects Act. (W16_154#16.178).

### Informed consent

Informed consent was obtained from all individual participants included in the study.

### Participants

We planned to include workers for a time frame of 11 months, from August 2016 until June 2017. In this period, a total of 1214 e-mail invitations were sent out by the occupational health service of an applied university, university, and academic hospital in The Netherlands to employees returning from sick leave or maternity leave of at least 2 weeks. The email invitations contained participant information and an informed consent form. After giving informed consent, the participants entered their contact information and date of RTW. Any personal or medical information of people that were invited for participation was not disclosed to the researchers, and the occupational health service received no information of participation, to preserve the privacy of the patients.

### Measurements and study design

The participants received three identical questionnaires at weeks 2, 5, and 8 after RTW, including an anchor question in the second questionnaire. The first questionnaire was sent out by email 2 weeks after the date of RTW. The second and third questionnaires were sent out 5 and 8 weeks after RTW, respectively. This questionnaire consisted of two parts. In the first part, the participant appraised their work ability on a scale of 0–10: 0 signifying no work ability at all, and 10 meaning the best work ability ever experienced by the participant. This is the first question of the Work Ability Index, also known as the Work Ability Score (WAS) (El Fassi et al. 2013; Gould et al. 2008). Work ability was sub-divided into general, physical, and mental/emotional work ability. The second part of the questionnaire consisted of the weighted composite work-functioning questionnaire (Boezeman et al. 2015b). This work-functioning questionnaire consists of 49 items spread over four domains: “Quality of work performance,” “Recovery from work,” “Quantity of work”, and “Capacity to work.” Scores for each of the domains were converted to a 0–100 score. Using these scores and a weighting factor, an end score representative of work functioning was calculated—also on a scale of 0–100—with a score of 0 meaning no problems with work functioning and a score of 100 meaning maximum limitation in work functioning (Boezeman et al. 2015a). At 5 weeks after RTW, the following anchor question was asked: did anything significantly change in the way you are able to carry out all your work satisfactorily since you returned to work? The three possible answers were: improved, not changed or deteriorated.

### Statistical analyses

Before the MIC calculation, the group averages on different time intervals after return to work were calculated and compared by means of a *T* test to describe the general improvement or deterioration of the participants over time.

To determine the relevant change in work ability and work functioning, the Minimal Important Change (MIC) was calculated for the study population. To put this in perspective of measurement error, we also calculated the Smallest Detectable Change (SDC). To answer the second research question, we determined the proportion of people who changed in their work ability and work functioning according to these statistical parameters.

First, the scores of the work ability questions and the work-functioning instrument were separated per answer on the anchor question. This separation was performed to distinguish between the different groups of people who can experience a different trajectory of work functioning and work ability, which may influence an overall average. This anchor question was used to determine the relevant change of the measurements. The MIC is defined by the COnsensus-based Standards for the selection of health Measurement INstruments (COSMIN) as the smallest change in score that is relevant to the patient (de Vet et al. 2011). The COSMIN checklist was used to improve the methodological quality of this study on measurement properties. The MIC was determined in two ways using the anchor-based method. These anchor-based methods use an external criterion, which is the relevant change as perceived by the patient/worker.

The first method we used to determine the MIC was the global ratings of change (GRC) or mean change method (Crosby et al. 2003). The mean change method was used to determine the difference between workers who experienced an improved work ability or work functioning and workers who reported that no change has occurred. In this method, the mean change score is calculated by first determining the individual change scores. The mean of the change scores of the group that has no improvement is subtracted from the mean of the change scores of the group that improved to find the difference. This difference represents the MIC based on the global ratings of change method. The same is done for the deteriorated and not changed groups to find the MIC of deterioration.

The second anchor-based method to determine the MIC is called the anchor-based MIC distribution (de Vet et al. 2007). This method assesses the MIC by a visual method integrating the Receiver Operating Characteristic (ROC) as recommended by de Vet et al. (de Vet et al. 2007). The MIC was defined as the optimal cut-off point on the ROC curve, closest to the upper left corner. The optimal cut-off point is the point with the minimal misclassification of patients (where the sum of the percentages of type 1 and type 2 error is the lowest) (de Vet et al. 2007). This point was calculated as the Youden’s J-statistic; Sensitivity + Specificity − 1 (Fluss et al. 2005). In the formula, the sensitivity and specificity are valued equally. This analysis was used to determine the optimal cut-off point of the area under the ROC curve (AUC) with sensitivity and specificity valued equally. The ROC plots the true-positive rate (sensitivity) against the false-positive rate (1 − specificity). Sensitivity is the chance of correctly classifying a change as being improved. The specificity is the chance of correctly classifying an individual as not changed (true negative rate). A larger AUC indicates a greater degree of correct classification. The AUC and the optimal cut-off point of the ROC were determined for both instruments in this study.

For both instruments, the SDC was calculated to check if the MIC was greater than the SDC (de Vet et al. 2006b). This SDC is based on measurement error is calculated with the formula: 1.96 × √(2) × SEM (de Vet et al. 2006a). The SDC is the smallest measurable change within a person that is not attributable to measurement error. As standards for acceptable AUC of ROC statistics, we used: values < 0.70 as inadequate, ≥ 0.7 to < 0.80 as acceptable, between ≥ 0.80 to < 0.90 as excellent, and ≥ 0.90 as outstanding (Hosmer and Lemeshow 2000). An AUC of at least 0.70 will be considered as adequate.

To answer the second research question, the change scores of the participants have been related to both MIC values and the anchor itself. These standards can be used to calculate the percentage of persons that have a change greater than the calculated cut-off points (van Kampen et al. 2013). In this way, it is possible to track the course of RTW. The percentage of participants who reported a positive change in RTW (according to the different MIC values) were calculated for each instrument and timepoint in this study. This analysis was done to evaluate the use of these two instruments in determining the percentages of workers who experience an increase in their work functioning or work ability in weeks 2–8 after RTW.

All analyses have been performed using IBM SPSS statistics 24. Global ratings of change and SDC were calculated using Microsoft Excel.

## Results

Of the 1073 e-mail invitations sent out by the occupational health service, a total of 308 people gave their informed consent. In total, 235 participants were eligible for participation in this research, after 73 people were excluded, because their date of RTW was over 2 weeks ago, or they did not return to their original workplace. A total of 170 persons completed the first questionnaire 2 weeks after RTW. Of the 170 participants, 85% were female, since we also invited people returning from pregnancy leave. A significant proportion (43%) of the population completing the questionnaire was over 51 years of age. The age categories 18–30, 31–40, and 41–50 constituted 10, 31, and 16%, respectively.

At weeks 5 and 8 following RTW, 161 and 155 people filled out the work ability questions, respectively. For 120 of these, it was possible to calculate a work-functioning problems score. The average baseline scores for work ability were 7.4, 7.6, and 7.3 for general, physical, and mental/emotional work ability, respectively, and 27.4 for work-functioning problems. The average baseline and follow-up scores are shown in Tables 1 and 2. Overall, although significant, the group scores barely differed in the early weeks after RTW.

**Table 1:** Mean group scores and standard deviations of work ability and work functioning in the early weeks after RTW

	T1 (2 weeks after RTW)	T2 (5 weeks after RTW)	T3 (8 weeks after RTW)
General work ability (0–10) (higher is better)	7.4 (1.28)	7.6 (1.26)*	7.6 (1.46)
Physical work ability (0–10) (higher is better)	7.6 (1.44)	7.8 (1.36)**	7.7 (1.40)
Mental/emotional work ability (0–10) (higher is better)	7.3 (1.66)	7.4 (1.52)	7.4 (1.52)
Work-functioning problems (0–100) (lower is better)	27.4 (16.39)	23.1 (16.27)*	26.1 (18.71)**

The number of workers is absent (*N* = 120–170)

*Sign. different from prior measurement (*P* < 0.01)

**Sign. different from prior measurement (*P* < 0.05)

**Table 2:** Mean and standard deviations for the three time intervals after RTW, grouped answer to the anchor question

	T1 (2 weeks after RTW)			T2 (5 weeks after RTW)			T3 (8 weeks after RTW)		
	*N*	Mean	SD	*N*	Mean	SD	*N*	Mean	SD
Deteriorated									
General work ability	13	6.5	1.05	14	5.9	1.49	11	5.5	1.92
Physical work ability	13	7.6	1.50	14	7.1	1.69	11	6.2	2.14
Mental/emotional work ability	13	6.5	1.76	14	5.9	2.06	11	5.4	1.57
Work-functioning problems	13	36.8	13.31	10	45.7	13.03	7	52.9	15.86
Not changed									
General work ability	70	7.5	1.33	82	7.7	1.19	59	7.6	1.29
Physical work ability	69	7.8	1.34	82	7.8	1.33	59	7.6	1.35
Mental/emotional work ability	70	7.4	1.67	82	7.3	1.52	59	7.2	1.61
Work-functioning problems	66	26.2	16.88	59	21.2	16.13	46	28.1	20.70
Improved									
General work ability	59	7.4	1.25	65	7.8	1.04	56	8.0	1.32
Physical work ability	59	7.5	1.47	65	7.9	1.29	56	7.9	1.32
Mental/emotional work ability	59	7.2	1.75	65	7.8	1.18	56	7.8	1.23
Work-functioning problems	59	28.1	16.88	51	20.8	13.65	46	20.6	14.52

On a group level, there are significant improvements on T2 compared to T1 for general and physical work ability, and for the work-functioning problems score. On T3, there was no significant difference in work ability compared to T2. The work-functioning score increases from T1 to T2 but decreases again on T3. This decrease was only significant for work functioning.

In Table 2, the scores of the respondents at 2, 5, and 8 weeks after return to work are shown. The respondents are grouped by their answer on the research question (deteriorated, not changed or improved on T2 when compared to T1) to give more insight to the trajectory of return to work. The three groups all experience a difference in their trajectory. The numbers at T2 are higher than the numbers at both other timepoints, because the file was split by answer on the anchor question. This anchor question was asked at T2. Therefore, it was possible to have missed the first (*N* = 28) or last (*N* = 21–29) measurement. If T2 was missed, it was not possible to classify the participant to one of the improved, not changed or deteriorated groups.

**Fig. 1:**
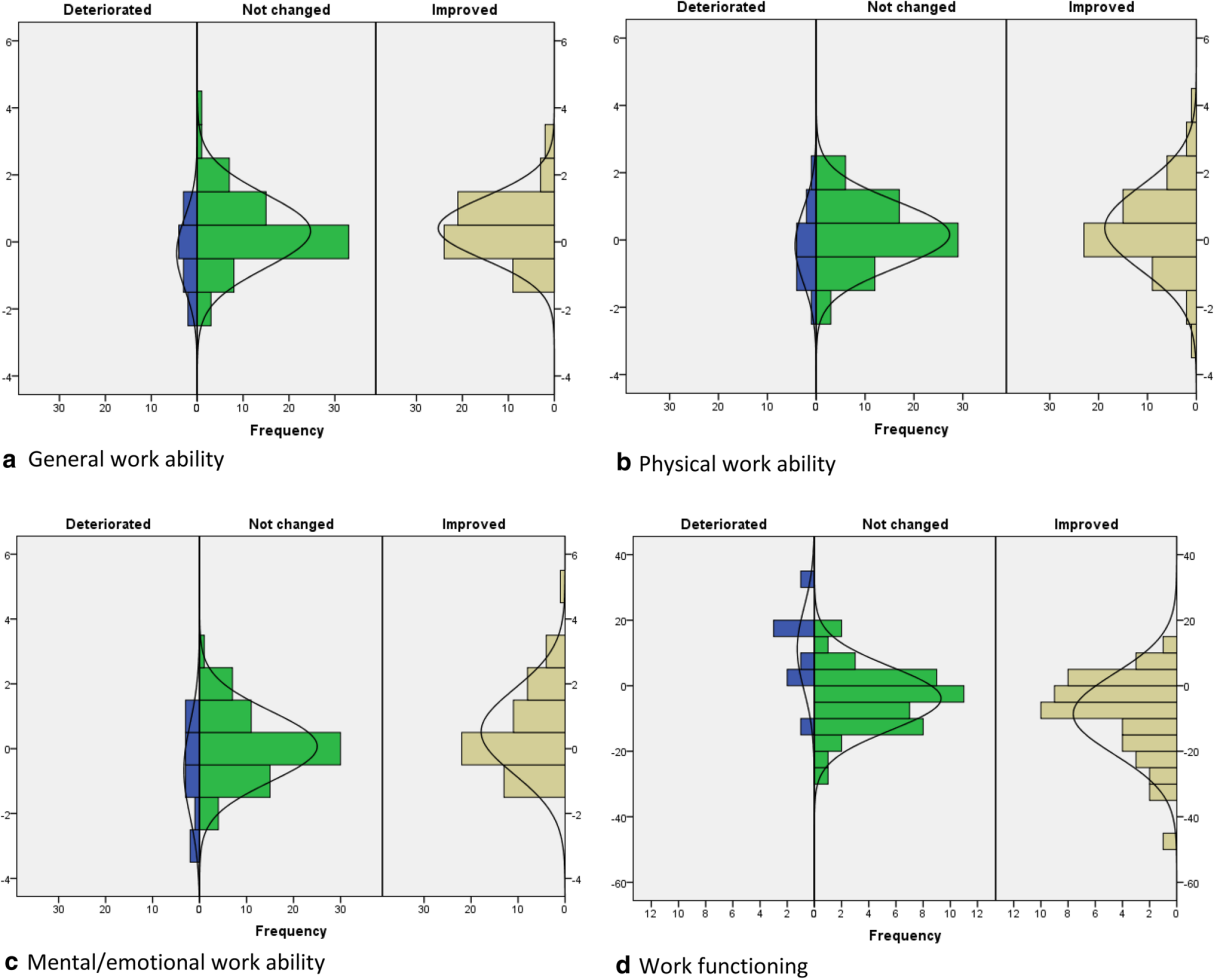
Distribution of change scores of work ability and work functioning (**a**–**d**), separated by answer on the anchor question (deteriorated, not changed and improved)

The first method used to calculate the MIC was the GRC method, using the group averages of persons, where a change score could be calculated. These values are shown in Table 3. The second method is the anchor-based MIC distribution method. As a first step, the visual representations of change scores are shown in Fig. 1a–d. These are the scores separated into the three groups deteriorated, not changed and improved, according to the anchor question.

**Table 3:** Minimal important change calculated in two ways and SDC of general, physical, and mental/emotional work ability and work functioning

	MIC based on GRC method		MIC based on ROC (AUC) method		SDC
	Improv.	Deter.	Improv.	Deter.	
General work ability	0.08	− 0.66	0.5	− 0.5	2.20
Physical work ability	0.19	− 0.33	0.5	− 0.5	1.93
Mental/emotional work ability	0.45	− 0.74	0.5	− 0.5	2.11
Work-functioning problems	− 4.77	15.07	− 5.96	3.47	19.90

*Improv* Improvement, *Deter* Deterioration

**Fig. 2:**
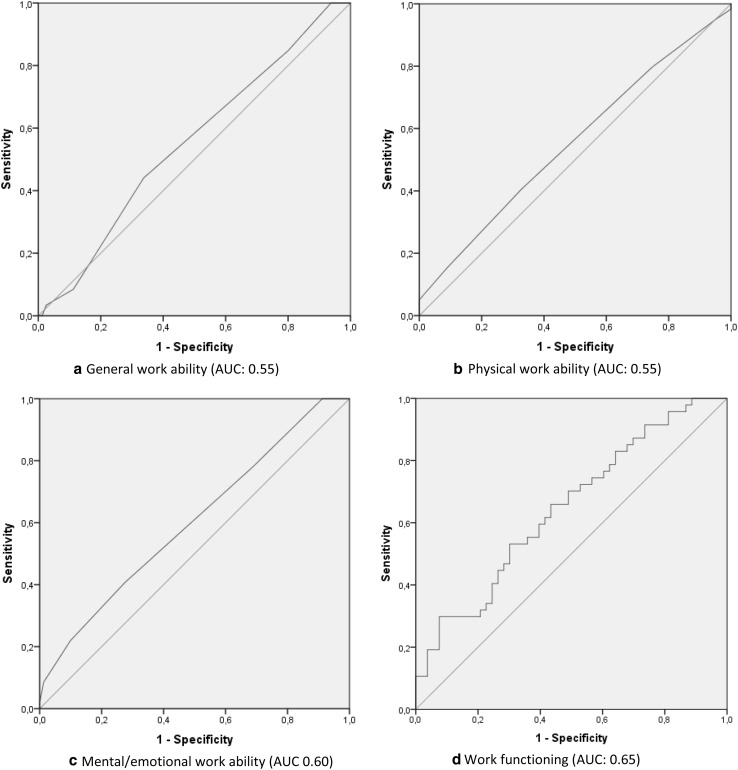
ROC curve with AUC values of improvement for work ability and work functioning

As part of the second step, the ROC curves for improvement are calculated and are shown in Fig. 2a–d. All AUCs of improvement were classified as inadequate. Although the AUC of deterioration (not shown) was higher than the AUC for improvement, only the AUC of work functioning was excellent with an AUC of 0.87. The AUCs for deterioration of general, physical, and mental/emotional work ability were 0.66, 0.61, and 0.66, respectively, which can be classified as inadequate (Hosmer and Lemeshow 2000).

The results of both MIC calculation methods for both improvement and deterioration, and the calculated SDC, are shown in Table 3. The scores for work functioning are inverted when compared to the work ability questions due to the scale of measurement. A higher score is positive for work ability but negative for work functioning. The point of minimal misclassification is the point closest to the upper left corner of the graph and is calculated using the Youden J-statistic (Fluss et al. 2005). The corresponding value to the highest J-statistic is shown in Table 3 as the MIC based on the AUC method.

As can be concluded from Table 3, there is effectively no difference between the GRC method and the ROC method for the working ability questions, because they are all smaller than 1 (or − 1). This is the minimum difference that a person can fill in on the questionnaire. According to the MIC calculated with both methods, 1 point difference signifies a relevant change with respect to a previous measurement. However, the SDC is over 2, meaning that the MIC falls within the range of measurement error. A change of one point on the work ability questions can thus still be attributed to measurement error.

The MIC for work-functioning problems calculated with the GRC method is − 4.77 for improvement and 15.07 for deterioration (due to the inverted scale of this instrument). When calculated with the ROC method, these values are − 5.96 and 3.47, respectively. However, both these values fall within the measurement error of the SDC, which is 19.90 point on a 100 point scale.

To assess if the SDC classifies the same amount of workers as changed (as according to the respondents’ answer on the anchor question), the calculated SDCs were used to classify participants in practice (Fritz et al. 2009). The percentage of workers that are improved or deteriorated according to the different SDC cut-off values is shown in Table 4. This table describes the percentages of participants that show improvement when the different SDC cut-off values for change are used. As shown, these cut-off values are conservative compared to the anchor question.

**Table 4:** Percentage of people deteriorated, not changed, and improved as classified by the different cut-off values as calculated in the first research question

	Change score T2–T1	T3–T2	T3–T1
	Changed between week 2 and 5	Changed between week 5 and 8	Changed between week 2 and 8
	% (*N*)	% (*N*)	% (*N*)
Anchor (only between T1 and T2)			
−	10 (16)	−	−
=	50 (84)	−	−
+	40 (67)	−	−
Total	100 (167)	−	−
SDC of general work ability WA (based on a SDC of 2 or larger)			
−	4 (5)	6 (7)	6 (7)
=	86 (120)	88 (111)	82 (102)
+	10 (14)	6 (8)	12 (15)
Total	100 (139)	100 (126)	100 (124)
SDC of physical work ability WA (based on a SDC of 2 or larger)			
−	5 (7)	6 (8)	8 (10)
=	83 (115)	86 (108)	80 (98)
+	12 (16)	8 (10)	12 (15)
Total	100 (138)	100 (126)	100 (123)
SDC of mental work ability (based on a SDC of 2 or larger)			
−	5 (7)	8 (10)	8 (10)
=	80 (111)	83 (105)	77 (95)
+	15 (21)	9 (11)	15 (19)
Total	100 (139)	100 (126)	100 (124)
SDC of work functioning (based on a SDC of 19.9 or larger)			
−	1 (1)	6 (5)	5 (5)
=	89 (89)	94 (85)	88 (84)
+	10 (10)	0 (0)	6 (6)
Total	100 (100)	100 (90)	100 (95)

− indicates deteriorated, = indicates not changed and + indicates improved according to the different SDC cut-off values

Eight weeks after RTW, 12–15% of the study population improved when the SDC of work ability is used as the cut-off point, compared to 2 weeks after RTW. When looked at the same statistic for work functioning, only 6% showed improvement. The percentage of people that improve after week 5 barely increases when looked at the SDC. A deterioration is more likely to occur between weeks 5 and 8 than between weeks 2 and 5.

## Discussion

### Key results

The responsiveness of three work ability questions and the work-functioning questionnaire in a sample of workers returning to work was inadequate. The workers that have improved cannot reliably be distinguished from persons that have not improved with these two instruments. Because the MIC values fall within the measurement error, the SDC values must be used to determine change (Rysstad et al. 2017; van Kampen et al. 2013). A change value above the SDC means that a relevant change has occurred that has a 95% certainty of not being attributable to measurement error. In this population, 10–15% showed a significant and measurable increase in work ability and work functioning over the first 5 weeks of RTW. Therefore, on a group level, work ability, and work-functioning increase minimally after RTW.

The responsiveness for deterioration is better than for improvement, but still inadequate, with the exception of the work-functioning instrument, which showed excellent responsiveness for the deteriorated group. However, it must be noted that this statistic was calculated using only 12 subjects; therefore, it must be interpreted with care. This is in line with the previous findings, where the AUC of the work ability question was found to be inadequate for distinguishing between persons at high or low risk for disability pension (Roelen et al. 2014). Another study by Abma et al. (2013)—studying the Work Role Functioning Questionnaire 2.0—showed similar results for their instrument (Abma et al. 2013). We found no instruments measuring similar constructs with excellent responsiveness.

Our assumption that people returning to work will have a lower Work Ability Score and a higher work-functioning problems score than the regular working population, which will then gradually return to normal values in the early weeks after RTW, seems only partly correct. This assumption was based on various models of RTW (Corrigan and McCracken 2005; Franche and Krause 2002; Hogelund et al. 2010). In these models, the employee is assumed to recommence work while at suboptimal working capacity. The goal in this phase is to let the worker gradually learn and function towards their full (new) capacity. These first weeks after RTW can be seen as the “action phase” (Franche and Krause 2002) or “re-entry phase” (Young et al. 2005), in which the worker recommences work and attempts to meet the workplace demands. This phase is seen as a critical phase in the RTW process because of the high risk for relapse (Franche and Krause 2002). Arends et al. (2014) state that analysis of work-functioning trajectories after RTW may help identify workers at risk for relapse and support these individuals to remain in work (Arends et al. 2014). In this study, we did not find a large increase in work ability or a substantial decrease in work-functioning problems over the first few weeks of RTW on an individual level. This may be due to the high baseline values (2 weeks after RTW). The average population in this study returned to work with values close to that of a normal working population. Compared to a normal working population (van Schaaijk et al. 2018), the baseline Work Ability Score was 0.7 lower, and the baseline work-functioning problems score was 10.2 higher, but the population returning to work does not reach the values of a normal working population in the first 8 weeks after RTW.

Although these work ability and work-functioning instruments are not suitable for determining change on an individual level, the scores at a group level showed that there is compliance with the described models. The minor increase in work ability and decrease in work-functioning problems at a group level at 5 weeks after RTW shows that the average population is coping with their work demands upon RTW (Table 1). The next step in a successful RTW is maintaining or even increasing work ability and work functioning. This phase is called “maintenance” or “stay-at-work” (Seing et al. 2015; Young et al. 2005). For a sustainable RTW, it is essential to detect workers, whose re-entry does not go as planned or workers who show a decrease in work ability and work functioning or who do not reach normal values. A decrease in these parameters, or not achieving the normal values of healthy working people, could be a reason for an occupational health professional to contact the worker with the aim of preventing a relapse. We found that 5–8% of the population in this study showed a decrease in work ability and increased work-functioning problems in the first 8 weeks after RTW. This population may be at risk of relapsing to sick leave again.

### Strengths and limitations

A strength of this study is that the anchor incorporates a meaningful change as a subjective outcome reported by the patient (McGlothlin and Lewis 2014). Another strength is that the population is followed over a time frame over which a change in work ability and work functioning could be expected. To our knowledge, this is the first study examining work ability and work-functioning trajectories in the first weeks after RTW. A drawback of this study is that a distinction between people returning from different causes of sick leave cannot be made. We chose to include all diagnoses, because we wanted the population to be heterogeneous and representative of the population of workers returning to work—thus, various scenarios resulting in an RTW are included. In addition, in The Netherlands, pregnancy leave falls within the scope of sick leave, and these individuals are, therefore, included in our analyses. Therefore, this study cannot make statements regarding specific populations or diagnoses. Another drawback is that a large number of participants who gave informed consent did not fill out the questionnaires at the different timepoints. It could be that the invitation to fill out the questionnaire has ended up in the spam filter. Considering this, the dropout over the three timepoints is minimal.

### Interpretation

The results of this study show that the responsiveness of the work ability questions and the work-functioning questionnaire is insufficient to determine a change in the early phase of RTW in this population of workers based on the MIC. The authors suggest to refrain from using the single item of the Work Ability Index, or the WAS score—for individual use, in this context, since the measurement error is high compared to the MIC.

Because the MIC is below the SDC, whenever a minimal important change occurs, this falls within the measurement error, meaning that it cannot be said with certainty that a relevant and measurable change has occurred. Therefore, although workers considered a small change as improved or deteriorated, the measurement error on this instrument makes it unable to detect this change. The SDC should thus be used as the cut-off value to conclude that a relevant and measurable change has occurred. For this reason, the SDC is used as the cut-off point to determine change in the second research question.

Large changes need to occur to be able to determine with certainty that a relevant, measurable change has occurred. With better instruments lacking, occupational professionals can use the SDC as a reference for change in populations returning to work—to determine a relevant and measurable change—bearing in mind that people not meeting this score can also experience an improvement or deterioration that falls within the measurement error. Only extreme cases will show up as improved or deteriorated. The best way for now to monitor the RTW process in a heterogeneous sample remains assessment by an occupational physician during a regular consultation, or a preventative medical examination. This underlines the complexity of monitoring work ability in (recently returned to work) workers.

Prior research has shown working people to generally appraise their work ability between seven and nine out of ten (de Zwart et al. 2002; El Fassi et al. 2013; Gould et al. 2008; Roelen et al. 2014; Schouten et al. 2016). A change of at least 2 for work ability and 20 for work functioning is hard to achieve and will rarely occur. Workers, therefore, can experience a change in these parameters without this being picked up by the instruments used. This factor can go some way to explain the large discrepancy between the number of people changed on anchor question and the appraised work ability or work functioning as presented in Table 4. Due to there being little variation in this population, the SDC is high compared to the MIC, making it hard to distinguish between people (as evidenced by the overlap in Fig. 1). Contributing to the low variation is the high baseline score at 2 weeks after RTW, signifying that workers returning to work at this point view themselves as being well capable of doing their job well, which is clearly good news.

Separating the scores over time by their answer on the anchor question sheds light on the reasons for the small increase in work ability and work functioning. The average scores change when the population is separated by the answer to the anchor question (Table 2). When workers grouped as decreased are excluded, and the analysis only includes people who score not changed and improved on the anchor question, it can be seen that scores do reach those of healthy workers around 8 weeks after RTW. This may indicate that the period after RTW is critical for occupational professionals to detect these people who are deteriorating and prevent these workers from falling back into sick leave again. This is an important group, and future research needs to focus on detecting people who are at risk of relapsing.

### Generalizability

Because the population studied are those returning to work after sickness, we expected it to have a larger variance in work ability and work-functioning scores than regular working populations. This variance makes it easier to distinguish persons from each other. However, even in this population, the majority of people score between a 7 and 9 on work ability (75% at T1). As expected, the increase in work ability and work functioning is significant on a group level, though only minimally. This increase directly after RTW plateaus towards week 8 after RTW as the total population scores are moving back towards the baseline score of 2 weeks after RTW. As hypothesized above, this may be attributable to the deteriorated group, who may have been signed off sick in a normal population, but remain in this population, because they have just returned to work. The calculated SDC and MIC may be generalizable to other heterogeneous populations in different workplaces, because the mean and standard deviation of the measurements in this study are known to be similar for different populations (de Zwart et al. 2002; El Fassi et al. 2013; Gould et al. 2008; Roelen et al. 2014; Schouten et al. 2016; van Schaaijk et al. 2018). However, it must be noted that in specific homogeneous populations, work ability and work-functioning scores and their variation among the study population can be different, resulting in different MIC and SDC scores, and therefore, different thresholds for increases or decreases for that specific population.

## Conclusion

Since the AUC for the work ability questions and the work-functioning instrument are inadequate, we advise refraining from using the single-item Work Ability Score to distinguish between individual workers who improve or deteriorate over the RTW process. The capacity of the work-functioning instrument to detect change in people returning to work is also inadequate, but better than the single-item work ability questions, especially for the deteriorated group. Because the MIC values of both instruments fall within that of measurement error, the SDC should be used to assess measurable, relevant change. This leads to the conclusion that 10–15% of the individuals in this study returning to work show an improvement greater than the SDC.
